# Characterizing Silence: Adolescents’ Nondisclosure of Their Suicidal Thoughts and Behaviors to Their Family and Peers

**DOI:** 10.1016/j.jaacop.2024.12.005

**Published:** 2025-02-20

**Authors:** Angela Page Spears, Ki Eun Shin, Christine B. Cha

**Affiliations:** a Teachers College, Columbia University, New York, New York; b Long Island University Post, Brookville, New York; c Yale School of Medicine, New Haven, Connecticut

**Keywords:** suicide, nondisclosure, adolescent, confidant selection

## Abstract

**Objective:**

Despite immense public concern around youth suicide, there remain countless young people whose suicide risk remain undetected. Some adolescents deliberately choose not to disclose suicide-related experiences, even to their family and peers who could play a key role in connecting them to clinical assessment and care. Studying this population is scientifically challenging, as those adolescents who are prone to nondisclosure are the exact individuals who may not typically opt into research studies. The current investigation explores the frequency, reasons for, and correlates of adolescents’ nondisclosure tendencies.

**Method:**

We recruited 154 adolescents (aged 13-17 years; mean = 15.98; SD = 1.04) with a lifetime history of suicidal thoughts and/or behaviors from across the United States to participate in a study requiring only adolescent self-consent. Adolescents were predominantly female (92.21%), White (72.08%), and bisexual (35.06%). We administered a brief, Web-based, anonymous survey assessing lifetime history of suicidal thoughts and behaviors, and nondisclosure of such suicide-related experiences.

**Results:**

Other family members of suicidal adolescents were the most common primary confidant identified to whom adolescents deliberately chose not to disclose their suicide-related experiences, closely followed by parents. Adolescents tended not to disclose to certain confidants because of their fear of negative reactions and sense of self-reliance. Those adolescents who had never disclosed to anyone tended to experience more frequent suicidal thoughts, to be closer with their primary confidants, and to cite distinct reasons for nondisclosure.

**Conclusion:**

Some adolescents remain silent about their suicide-related experiences, either toward select confidants or all of the people in their life. Understanding the distinct profiles of and reasons for nondisclosure may inform ways to connect underserved youth to care.

**Diversity & Inclusion Statement:**

We worked to ensure that the study questionnaires were prepared in an inclusive way. We worked to ensure race, ethnic, and/or other types of diversity in the recruitment of human participants. We worked to ensure sex and gender balance in the recruitment of human participants. One or more of the authors of this paper self-identifies as a member of one or more historically underrepresented racial and/or ethnic groups in science. One or more of the authors of this paper self-identifies as a member of one or more historically underrepresented sexual and/or gender groups in science. One or more of the authors of this paper self-identifies as living with a disability. We actively worked to promote inclusion of historically underrepresented racial and/or ethnic groups in science in our author group. The author list of this paper includes contributors from the location and/or community where the research was conducted who participated in the data collection, design, analysis, and/or interpretation of the work.

## Plain language summary

Some adolescents experiencing suicidal thoughts or behaviors choose not to share them with certain family members, peers, or even anyone in their lives. This study surveyed 154 adolescents across the United States who self-reported a history of suicidal thoughts or behaviors to explore why they might not disclose these experiences. Adolescents often avoided disclosing to parents or other family members due to fears of negative reactions or a preference to cope on their own (ie, self-reliance). Those who disclosed to no one reported more frequent suicidal thoughts and closer relationships with their confidants. Understanding these patterns of nondisclosure may help connect underserved youth to care.

When adolescents engage in conversations with friends, romantic partners, and family they are faced with critical choices about what to reveal and what to hold back. Choosing not to make one’s thoughts known to family and peers (ie, nondisclosure) may result in a cascade of consequences including persistent rumination, social isolation, and a diminished sense of connection.^1^, ^2^, ^3^ When it comes to risk of suicide death among adolescents, these consequences are heightened, with psychological effects of nondisclosure to supportive figures linked to an elevated risk of future suicidal thoughts and attempts.^4^^,^^5^ Nondisclosure to family and peers may also hinder adolescents’ treatment access, as most suicide prevention efforts rely on social contacts to facilitate or advocate for intervention.^6^

Insights into nondisclosure tendencies can be drawn from other areas of research in ways to inform nondisclosure of suicide-related experiences. Studies on child sexual abuse survivors and lesbian, gay, bisexual, transgender, and queer/questioning (LGBTQ+) individuals have shown that people selectively disclose based on specific risks and benefits.^7^^,^^8^ Thus, nondisclosure also has an adaptive potential. For example, nondisclosure to family and peers may protect individuals from emotional distress or conflict.^9^ The varied nature of nondisclosure to family and peers also highlights that it is not merely a binary event (eg, disclosure vs nondisclosure); rather, they both have their own motivations and outcomes, demonstrating that nondisclosure is not just the absence of disclosure, but a separate phenomenon.^7^^,^^8^ Within nondisclosure to family and peers, there are a multitude of patterns that are affected by the interplay of individual, relational, and contextual factors.^10^ These patterns underscore the concept of selective outness, or selective nondisclosure, whereby individuals strategically share information based on personal reasons and specific contexts.^11^

Similar patterns have been identified in nondisclosure of suicidal ideation corresponding with confidant availability, the context in which suicide-related experiences occur, and personal motivations such as avoiding hospitalization.^12^, ^13^, ^14^ Depending on how these factors appear, adolescents may show varied patterns of not disclosing to one confidant while choosing to disclose to another (ie, selective nondisclosure). However, this captures only some adolescents who are not disclosing their suicide-related experiences. In a study by Fox *et al.*, 22% of adolescents with suicidal thoughts and 27% with a history of suicide attempts did not disclose to any confidant.^15^ These adolescents who chose to disclose to no one at all would be identified as perpetual nondisclosers. This subset of adolescents who forgo any support related to their suicide-related experiences is particularly concerning, as they may miss out on the emotional support that family and friends can provide in response to this kind of distress.^16^ Understanding and addressing both selective and perpetual nondisclosure are crucial for supporting these adolescents.

In recent years, research on the nondisclosure of suicide-related experiences and the resulting risks of adolescents remaining undetected and untreated has focused on the examination of nondisclosure to their clinicians.^15^^,^^17^ McGillivray *et al.* found that even among youth with access to a mental health provider, 39% never disclose their suicidal ideation to their providers.^17^ Similarly, Fox *et al.* found that adolescents with a history of mental health treatment were more likely to disclose their nonsuicidal self-injury, suicidal ideation, and suicide attempts when asked directly, but 17% to 36% still did not share these with their mental health providers.^15^ Common barriers to suicide-related disclosure identified in these studies include fears of losing autonomy, informing parents, and hospitalization.^15^^,^^17^ In addition, adolescents seem to prioritize talking about other mental health issues with their providers, such as anxiety or depression.^15^ The quality of the therapeutic relationship also seems to play a role, as adolescents choose not to disclose if they lack strong rapport with their provider or for fear of embarrassment and judgment.^18^^,^^19^ Not only do these findings suggest that there are many reasons why adolescents may choose not to disclose, but also they reveal how nondisclosure operates as a distinct phenomenon with its own set of motivations. These reasons for nondisclosure to clinicians differ from reasons for disclosure, such as the need for emotional support or understanding.^18^

Understanding whom adolescents rely upon during critical moments when health care professionals are unavailable is equally crucial. Fox *et al.* found that adolescents reported most frequent disclosure of all self-injurious thoughts and behaviors to their friends.^15^ This aligns with broader research indicating that young people are more likely to disclose their suicidal ideation to family and friends than to clinicians.^20^ Although many adolescents turn to their family, friends, or romantic partners for help, there remain some who withhold their suicide-related experiences from some or all of these confidants. Thus, examining close individuals within an adolescent’s life to whom they often turn (ie, primary confidants), but to whom they do not disclose their suicide-related experiences, may reveal distinct reasons behind nondisclosure.^21^

There are 4 key knowledge gaps pertaining to adolescents’ nondisclosure of suicide-related experiences. First, most studies focus on disclosure, overlooking nondisclosure as a distinct phenomenon with unique motives such as perceived judgment, fear of hospitalization, or concerns about therapy.^12^^,^^22^ Second, examining nondisclosure among youth is challenging because of parental consent requirements, which has rarely been overcome by requiring only self-consent (vs parental consent).^15^ Third, research has identified general disclosure patterns, but has not differentiated between perpetual and selective nondisclosures, which is important for understanding why some adolescents consistently avoid seeking any support.^23^ Fourth, specific reasons for nondisclosure to primary confidants remain unclear, with previous research focusing on barriers to disclosure to mental health professionals.^17^

To address these knowledge gaps, in the current study we aimed to investigate the following: (1) rates of suicide-related nondisclosures among adolescents to family and peers; (2a) motivations behind adolescents’ decisions to withhold suicide-related information from their primary confidants; (2b) the extent to which reasons for nondisclosure differ across primary confidant types (eg, parents vs other family vs friends vs significant others); and (3) correlates of the decision to disclose to anyone in their life or not (eg, perpetual nondisclosers vs selective nondisclosers).

## Method

### Sample

Adolescents across the United States were recruited via Instagram advertisements in 2021 to “help researchers understand adolescents’ experiences revealing their suicidal thoughts and behaviors.” Interested adolescents accessed an anonymous survey through a provided link, where they were told “At no time during this pre-screen will we ask questions that will identify you.” The pre-screen screened for eligibility and included the following: (1) endorsing at least 1 of 3 items (eg, “Have you ever had thoughts of killing yourself?”; “Have you ever thought of a way or method to kill yourself?”; “Have you ever tried to kill yourself?”) adapted from the Self-Injurious Thoughts and Behavior Interview**–**Revised (SITBI-R); (2) being 13-17 years of age; (3) having Internet access; (4) being proficient in English.^24^^,^^25^ Exclusion criteria were refusal to provide informed assent or current residence outside the United States. Our initial sample comprised 200 completed surveys. To ensure data validity, we excluded 6 potential bot responses and 3 duplicate submissions flagged by Qualtrics. All participants demonstrated responses within the expected accuracy range on attention-check questions, so no exclusions were made on this basis.^26^

Seven adolescents who selected “NA—no one in my life” when asked about confidants were excluded, as the study aimed to explore the decision of nondisclosure in the context of having people to whom these individuals could disclose. In addition, 30 adolescents who disclosed to everyone in their lives were excluded to focus on varying degrees of nondisclosure, and because those who disclosed to everyone did not have a primary confidant whom they did not disclose for us to ask them about.

The final sample included 154 adolescents (mean age = 15.98 years; SD = 1.04; range = 13-17) predominantly female (92.21%), White (72.08%), bisexual (35.06%), and middle class (mean = 5.42; SD = 1.48). Table 1 provides a breakdown of demographic characteristics.

**Table 1:** Demographic Characteristics of Sample

	Total sample (%)	Perpetual nondiscloser (%)	Selective nondiscloser (%)
Gender identity			
Male	7.14	6.98	7.21
Female	55.19	67.44	50.45
Male transgender	7.79	4.65	9.01
Nonbinary	20.78	18.60	21.62
Unsure	5.84	0	8.11
Other	3.25	2.33	3.60
Sexual orientation			
Heterosexual	21.43	18.60	22.52
Homosexual	13.64	20.93	10.81
Bisexual	35.06	32.56	36.04
Questioning	11.04	11.63	10.81
Prefer not to say	0.65	0	.90
Unsure	2.60	0	3.60
Other	15.58	16.28	15.32
Race			
Asian	11.69	16.28	9.91
Black/African American	14.94	20.93	12.61
Native American/ Alaska Native	5.84	4.65	6.31
Native Hawaiian/Pacific Islander	1.30	2.33	.90
Other	7.14	6.98	7.21
White	72.08	55.81	78.38
Ethnicity, Hispanic	19.48	13.95	21.62
Part-time employment	34.42	37.21	33.33

In their lifetime, most adolescents (78.57%) reported nonsuicidal self-injurious behavior, whereas all (100%) endorsed passive suicidal ideation (eg, thoughts about wanting to die without specific plans or intent to act), and most (70.13%) reported active suicidal ideation (eg, thoughts about wanting to die with specific plans or intentions to act). Hospitalization because of suicidal ideation occurred in 14.94% of cases. In addition, 87.01% had contemplated a suicide plan with a specific method, and 37.66% had attempted suicide, with 24.14% requiring hospitalization for these attempts.

### Procedure

A comprehensive account of the recruitment, screening, and study procedures can be found in a forthcoming work (Spears *et al.*, unpublished, 2025). All research protocols were granted approval by the Institutional Review Board (#21-286) at Teachers College, Columbia University, and followed the recently published best practice guidelines for online research on adolescent self-injury.^27^ Adolescents who met the necessary criteria and gave their assent completed a true**–**false quiz to check for understanding. They were then granted access to the complete study through Qualtrics, with parental consent being waived similar to previous work.^15^^,^^28^, ^29^, ^30^ Every individual who completed the initial screening or the full study was offered mental health support resources. Furthermore, participants who finished the full study were offered a $10 Starbucks gift card.

### Measures

#### Demographic Information

All demographic information, as presented in Table 1, was collected via a self-report questionnaire. Socioeconomic status was evaluated using the MacArthur Scale of Subjective Social Status**–**Youth Version.^31^

#### Past Suicide-Related Experiences

The Self-Injurious Thoughts and Behavior Interview**–**Revised (SITBI-R) assessed lifetime suicidal thoughts, suicide plans, and suicide attempts.^24^^,^^25^ Follow-up questions gathered more details if endorsed including frequency (ie, suicidal ideation [“Thinking about all the times that you have had these thoughts, on average, how frequently did you have these thoughts?”], suicide plans [“On average, how frequently have you thought of a specific method, a specific place, or a specific time for your suicide plan(s)?”]), severity, and future likelihood). This measure maintains strong validity and reliability.^24^^,^^25^

#### Primary Confidant Selection

To identify the one primary confidant to be examined in this study, adolescents were asked a series of questions. Adolescents were provided with a list of family members and peers including mother, father, sister, brother aunt, uncle, cousin, grandmother, grandfather, family friend, friend, acquaintance, and partner/lover. These were selected by the authors in 2 ways. First, the authors identified confidants from previous research on adolescent disclosure of suicidal thoughts and behaviors, such as parents (ie, mother and father), friends (eg, friend and acquaintance), and romantic partners (eg, partner/lovers).^22^^,^^32^ Second, to build on previous research on disclosure, the authors also drew from studies of how extended family members, or nonparental adults, influence adolescent well-being to examine other family members (eg, sister, brother, aunt, uncle, cousin, grandmother, grandfather, and family friend).^33^ Adolescents were provided this list and asked to select all social contacts who were present in their lives. Next, from the list of family and peers that adolescents had indicated as a part of their lives, adolescents selected one confidant with whom they most frequently shared intimate information (ie, primary confidant), but to whom they had not disclosed their suicide-related experiences. Closeness to that primary confidant was measured by the Unidimensional Relationship Closeness Scale (URCS).^34^

#### Reasons for Nondisclosure

We developed and administered the Reasons for Suicide-Related Nondisclosure Questionnaire (R-SNDQ) to explore the factors influencing suicide-related nondisclosure (Supplement 1, available online). Questions were informed by earlier studies that analyzed barriers to disclosure among adults.^18^^,^^35^ Adolescents rated their responses on a 5-point Likert scale, ranging from 0 (not at all) to 4 (extremely). In our study, the R-SNDQ demonstrated robust internal consistency (α = 0.78-0.86).

### Data Analysis

Data screening and statistical analyses were conducted using IBM SPSS version 29 for Mac. Factor analyses were performed using R Studio version 4.2.1 with the *psych* and *lavaan* packages.

#### Aim 1: Rates of Suicide-Related Nondisclosures Among Adolescents to Family and Peers

To address aim 1, we calculated descriptive statistics including frequencies for the types of disclosure behaviors (perpetual nondisclosers vs selective nondisclosers) and the distribution of primary confidants within these groups.

#### Aim 2a: Motivations Behind Adolescents’ Decisions to Withhold Suicide-Related Information From Their Primary Confidants

Suitability for factor analysis in aim 2 was checked by assessing distribution items based on skewness, kurtosis statistics, the Bartlett test of sphericity, and the Kaiser**–**Meyer**–**Olkin measure of sampling adequacy. To address aim 2a, we conducted an exploratory factor analysis (EFA). A robust weighted least-squares estimator was used and yielded the best-fitting factor solution for the R-SNDQ. This estimation method has been shown to perform well in small samples and in the presence of nonnormality based on prior simulation studies.^36^ Multiple indices were considered to determine the appropriate number of factors, including the scree plot, parallel analysis, factor interpretability, and model fit. Model fit was determined based on a root mean square error of approximation (RMSEA), standardized root mean square residual (SRMR), and Tucker–Lewis index (TLI).^37^^,^^38^ Oblique oblimin rotation was used in each EFA. Interpretation of the factor structure was guided by examining item loadings on factors. Items with no sufficient loadings across factors (<0.40) or high cross-loadings (>0.40) were removed. The internal consistency of these summary measures was assessed using the Cronbach alpha. A minimum value above 0.70 for the Cronbach alpha indicated satisfactory internal consistency levels.

#### Aim 2b: The Extent to Which Reasons for Nondisclosure Differ Across Primary Confidant Types (eg, Parents vs Other Family vs Friends vs Significant Others)

To address aim 2b, we conducted independent-samples Kruskal**–**Wallis tests.

#### Aim 3: Correlates of the Decision to Disclose to Anyone in Their Life or Not (eg, Perpetual Nondisclosers vs Selective Nondisclosers)

Before conducting inferential statistics in Aim 3, assumptions regarding normality were evaluated by performing a Shapiro**–**Wilk test on continuous variables. To address aim 3, we conducted independent-samples Mann**–**Whitney *U* tests and Fisher exact tests.

## Results

### Rates of Suicide-Related Nondisclosure

Among adolescents who reported having at least one available social contact, 27.92% refrained from making any suicide-related disclosures to family or peers currently in their lives (ie, perpetual nondisclosers). Most adolescents in our sample (72.08%) had disclosed to a confidant, but identified at least one social contact who was available to them, but to whom they chose not to disclose (ie, selective nondisclosers).

Adolescents could endorse only one category (eg, mother) as their primary confidant. Across all nondisclosing adolescents (perpetual and selective), the following primary confidants were chosen: 34.42% indicated a parent (eg, mother or father), 35.06% indicated another family member (eg, sister, brother, grandmother, grandfather, aunt, uncle, or cousin), 4.55% indicated their significant other, and 25.97% indicated their friend. Youth could endorse only one category as their primary confidant.

### Reasons for Suicide-Related Nondisclosure

**Figure 1:**
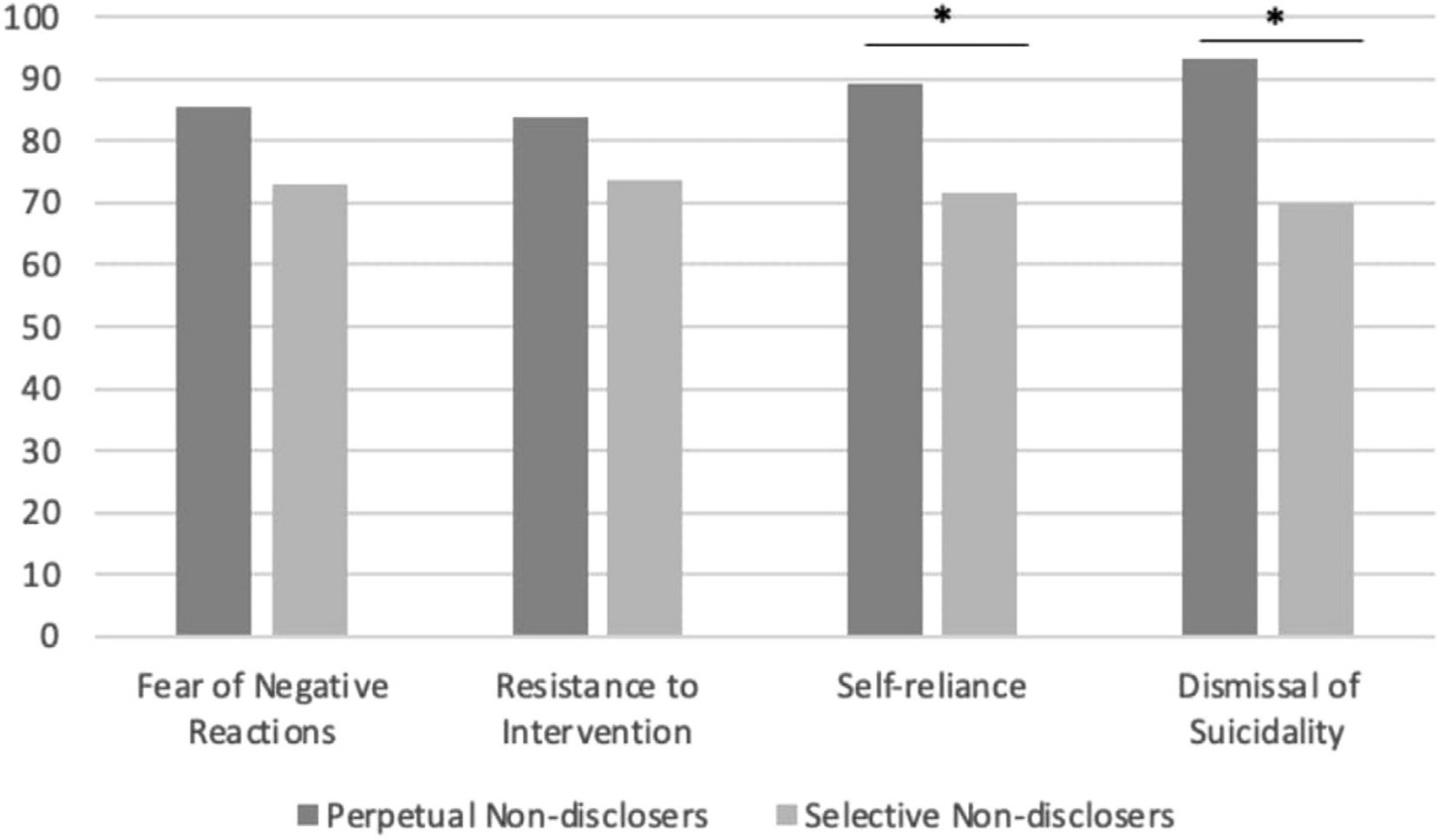
Mean Ranks of Nondisclosure Reasons: Perpetual vs Selective ***Note:****Perpetual nondisclosers have disclosed to no one in their lives, whereas selective nondisclosers have disclosed to some people in their lives but not others.*

Perpetual and selective nondisclosers provided a range of reasons why they chose not to disclose to their primary confidant. Taken together, results of the EFA yielded a 4-factor solution including fear of negative reactions (n = 3 items), resistance to intervention (n = 3 items), dismissal of suicidality (n = 2 items), and self-reliance (n = 4 items). Factor loadings can be found in Table 2. The KMO index for sampling adequacy indicated suitability for factor analysis (0.80).^39^ The Bartlett test of sphericity also indicated sufficient correlations between items on the measure (*p* values <.001). The scree plot suggested a 5-factor solution. However, based on parallel analyses, 5 factors showed eigenvalues higher than would be expected in random data. In addition, the factor pattern and correlations, model fit, and item content were considered in evaluating the factor structure. Between 5-factor and 4-factor solutions, a 4-factor solution led to better model fit, greater number of factors with at least 3 items with strong loadings (>0.40), and enhanced factor interpretability. Four items did not exhibit strong loadings with any factors (“I wasn’t ready to face my suicidal thoughts”; “I didn’t want them tell someone else”; “I did not feel comfortable sharing such intimate information”; “I have not had the chance to tell them yet”). These items were removed, and EFAs were conducted on the remaining 12 items. This indicated overall good model fit (TLI = 0.96, RMSEA = 0.14, SRMR = 0.04) and explained 77% of the total variance. Adolescents endorsed self-reliance (mean = 2.64; SD = 1.19) and fear of negative reactions (mean = 2.41; SD = 1.34) as reasons for nondisclosure most often followed by resistance to interventions (mean = 1.49; SD = 1.24) and dismissal of suicidality (mean = 1.00; SD = 1.26) (Figure 1). The fear of negative reactions factor was significantly correlated with the resistance to intervention (*r* = 0.45), self-reliance (*r* = 0.53), and dismissal of suicidality factors (*r* = 0.22) (*p* values <.05). The resistance to intervention factor was also significantly correlated with the self-reliance factor (*r* = 0.30, *p* < .001). There was no significant correlation between the resistance to intervention and dismissal of suicidality factors (*r* = 0.10), or between the self-reliance and dismissal of suicidality factors (*r* = 0.16) (*p* values >.05). Internal consistency was good across factors: fear of negative reactions (α = 0.86), resistance to intervention (α = 0.78), self-reliance (α = 0.83), and dismissal of suicidality (α = 0.82).

**Table 2:** Exploratory Factor Analysis Results for the Reasons for Suicide-Related Nondisclosure Questionnaire (R-SRNDQ)

Item	Factor 1: fear of negative reactions	Factor 2: resistance to intervention	Factor 3: self-reliance	Factor 4: dismissal of suicidality	Mean (SD)
I was afraid they would judge me.	**0.91**	0.08	–0.05	0.06	2.53 (1.50)
I was worried that they would be angry with me.	**0.77**	0.11	–0.09	0.06	2.07 (1.60)
I was worried that they would think of me differently.	**0.75**	0.07	0.23	–0.04	2.63 (1.45)
I was afraid of being forced into treatment I didn’t want.	0.07	**0.95**	0.06	–0.04	1.76 (1.57)
I was afraid that I would be hospitalized.	0.14	**0.87**	0.01	–0.03	1.86 (1.56)
I did not want them to stop me from my future suicide plans.	–0.01	**0.47**	0.03	–0.004	0.86 (1.33)
I did not want to worry them.	0.14	–0.24	**0.80**	–0.004	3.17 (1.27)
I did not want to be viewed as weak.	–0.06	0.26	**0.79**	0.13	2.36 (1.54)
I did not want to be viewed as unstable.	–0.003	0.30	**0.77**	0.13	2.61 (1.46)
I did not want it to damage our relationships.	0.43	–0.12	**0.60**	0.01	2.41 (1.56)
I will not experience suicidal thoughts and behaviors in my future so there was no point in disclosing;	–0.02	0.007	–0.05	**0.97**	0.86 (1.27)
it is not part of who I am now.	0.11	–0.13	0.11	**0.81**	1.14 (1.46)

Note: Factor loadings represent rotated factor loadings. Boldface type indicates loadings are retained for each factor.

Further examination of reasons for nondisclosure across perpetual and selective nondisclosers revealed that resistance to intervention was the only reason that significantly differed by primary confidant type (*H* = 9.47; *p* = .02). However, after adjusting for multiple tests, no pairwise comparisons remained significant (*p* values >.05).

### Correlates of Perpetual vs Selective Nondisclosure

Although 2 reasons for nondisclosure (ie, fear of negative reactions and resistance to intervention) did not differ between perpetual and selective nondisclosure (*p* values >.05), self-reliance (*U* = 1793; *p* = .02) and dismissal of suicidality (*U* = 1624; *p* = .002) significantly differed between groups. Perpetual nondisclosers reported self-reliance (mean rank = 89.30) and dismissal of suicidality (mean rank = 93.23) as greater reasons for nondisclosure to their primary confidant compared to selective nondisclosers (mean rank = 71.45 and 69.90, respectively).

Lifetime frequency of suicidal thoughts differed between types of nondisclosers, with perpetual nondisclosers reporting more frequent suicidal thoughts (mean rank = 89.51) compared to selective nondisclosers (mean rank = 72.85; *U* = 1870.00; *p* = .03). However, lifetime frequency of making suicide plans (*U* = 1656.00; *p* = .39) and number of lifetime number of suicide attempts (*U* = 165.50; *p* = .43) did not differ significantly.

Type of primary confidant selected did not differ depending on perpetual vs selective nondisclosers ([3, n = 154] = 4.10, *p* > .05, φc = 0.17). In addition, selective nondisclosers’ primary confidants did not differ significantly from the primary confidants to whom they had chosen to disclose ([9, n = 111] = 8.77, *p* > .05, φc = 0.16). Closeness to primary confidants revealed that perpetual nondisclosers reported more closeness to their primary confidant (mean rank = 92.87) than selective nondisclosers (mean rank = 71.55; *U* = 1725.50; *p* = .008).

## Discussion

This study is among the first to examine patterns of nondisclosure of suicide-related experiences among adolescents to family and peers. By waiving parental consent and ensuring anonymity, we addressed barriers that might have prevented participation, particularly for adolescents hesitant to disclose their suicide-related experiences. We found that although most adolescents tend to selectively disclose their suicide-related experiences to certain family members or peers, a distinct group of perpetual nondisclosers never share these experiences with anyone. This study highlights the differences between selective and perpetual nondisclosers in terms of their reasons for nondisclosure, the frequency of their suicide-related thoughts, and the nature of their relationships with their closest confidants.

More than a quarter of nondisclosing suicidal adolescents, despite having available primary confidants, did not disclose their suicide-related experiences to anyone in their life. These results align with findings from Fox *et al.* that indicated that adolescent nondisclosure rates to mental health professionals, parents, and peers was 22% to 27%.^15^ This research builds on previous work by further identifying a group of selective nondisclosers who have disclosed to some people in their lives but not others. A recent scoping review by Davies *et al.* highlights that there may be different barriers to disclosures related to different confidants.^20^ To our knowledge, this is the first paper that has identified confidants to whom adolescents do not disclose their suicide-related experiences, despite sharing other personal or “intimate” information. Among selective and perpetual nondisclosers, the percentages of primary confidants to whom adolescents have not disclosed their suicide-related experiences are relatively similar across categories (25.97%-35.06%), with the exception of romantic partners (4.55%). However, among those who disclosed (ie, selective nondisclosers and those who disclosed to everyone in their lives), 17.02% reported their romantic partners as their primary confidant and also disclosed to them. This finding aligns with prior literature among adults that indicate romantic partners as the first or only individuals in a suicidal person’s network to learn about the crisis.^40^ This finding is also supported by research in adolescent development, as it is possible that adolescents with romantic partners more often disclosed to them because of the intimacy of these relationships.^41^

This study additionally revealed distinct variations between selective and perpetual nondisclosers. Perpetual nondisclosers reported more frequent lifetime suicidal thoughts. One potential reason for this is that the lack of disclosure prevents them from accessing necessary support that could help decrease their suicidal thoughts. Previous research has emphasized the challenges of perpetual nondisclosure, highlighting potential implications for limited access to support.^16^ This is particularly true among adolescents with suicide-related experiences, as parents often serve as gatekeepers for mental health treatment and may be better positioned than peers to connect an adolescent with care; however, they often do not know that an adolescent is experiencing suicidal thoughts until they disclose.^6^ This highlights the potential helpful nature of selective nondisclosure, as it may be adaptive for adolescents to seek support from friends and romantic partners related to other topics, while deciding not to disclose their suicide-related experiences. Results also indicated that perpetual nondisclosers felt closer to their primary confidants than selective nondisclosers. These results could be interpreted through the interpersonal theory of suicide, which proposes that suicidal desire stems from feeling like a burden to others (ie, perceived burdensomeness) and feeling socially disconnected (ie, thwarted belongingness).^4^ Perpetual nondisclosers may feel closer to their primary confidants, or may have less thwarted belongingness, but this closeness may go hand in hand with increased feelings of perceived burdensomeness. It is possible that feeling closer to their primary confidants may increase feelings of being a burden, as perpetual nondisclosers may want to protect their primary confidants from worry or distress.

Adolescents’ primary reasons for nondisclosure of their suicide-related experiences to their primary confidant were self-reliance and fear of negative reactions. In other words, adolescents chose not to disclose their suicide-related experiences to someone with whom they often shared personal information, so as to preserve their own image or the relationship. These results are similar to previous research on culturally minoritized adolescents and their reasons for nondisclosure, where we found that racially minoritized adolescents reported significantly higher concerns about negative reactions compared to their White counterparts.^42^ Hom *et al.* identified similar barriers to disclosure to family members and friends including fear of worrying them, embarrassment, and being judged.^18^ However, there has been limited research to date on reasons for nondisclosure of suicide-related experiences among adolescents. When examining nondisclosure of suicide-related experiences among young adults (eg, people in their 20s), researchers have found similar results such as fear of negative reactions and confidants having had unhelpful reactions in the past.^13^^,^^43^ One area in which these reasons for nondisclosure diverge from findings by Shin *et al.* were that in their study, racially minoritized nondisclosing adolescents also more strongly endorsed a resistance to intervention than did their White counterparts.^42^ This may stem from a mistrust of mental health systems among racially minoritized groups. Prior studies have recognized multiple reasons for adolescent nondisclosure to clinicians; however, our research represents an initial quantitative exploration of these specific factors within adolescents’ nondisclosure to family members and peers. Similar to past studies, fear of hospitalization was identified as a reason for nondisclosure.^15^^,^^17^ Yet, despite being focused on as a primary reason for nondisclosure in past studies, it is notable that it was not in the top 2 most important reasons for these adolescents in relation to their primary confidants. This may be because the potential repercussions would be more interpersonal and understood in terms of loss of emotional support.

Seven notable constraints warrant consideration when interpreting the findings of this study. First, the predominant representation of White female adolescents within the sample might restrict the broader applicability of the results to diverse demographic cohorts. Given that the study was conducted with a predominantly White sample, it is important to consider the cultural significance of self-reliance and fear of negative reactions in the context of nondisclosure. In Western cultures, which often emphasize individualism, self-reliance can be seen as a culturally reinforced value, potentially discouraging adolescents from seeking help when experiencing suicidal thoughts.^44^ The hesitation to disclose may reflect broader cultural expectations around managing personal struggles independently, prioritizing self-sufficiency over collective support.^45^ Second, the study’s smaller sample size limited insights into nondisclosure among romantic partners. Although the robustness of broader patterns observed among primary confidants instills confidence in the study’s findings, in cases where multiple comparisons were made, this may have led to loss of significance. Third, the focus on nondisclosures to adolescents’ family and peers within this study limited exploration of clinicians, school personnel, and anonymous nondisclosures on social media. This choice was guided by the significant role that close, in-person relationships play in suicide prevention, as family and friends are typically positioned to offer direct support and intervention.^46^ Future research could expand by examining how adolescents engage in anonymous disclosures on social media platforms, where anonymity may reduce fears of judgment.^47^ Fourth, the exclusion of fully disclosing adolescents, because of the measurement requiring a confidant to whom they did not disclose, limited comparisons with full disclosers. Including them could clarify differences between disclosure and nondisclosure motivations.^18^ Fifth, the recruitment approach via Instagram limited exploration of youth who may be less active online or may not have access to electronic devices.^48^ Sixth, the lack of transparency regarding Instagram’s advertisement targeting algorithms could introduce sample bias. In particular, if Instagram showed ads to certain groups of adolescents more than others (eg, based on engagement with mental health content), this could have led to a nonrepresentative sample.^48^ Seventh, the inclusion of suicide terminology may have also limited the participation of some nondisclosing youth. The explicit mention of suicide in the advertisement and initial questions likely attracted participants more comfortable with the topic, potentially excluding those who avoid discussing because of fear or discomfort.^49^

Although past research has often emphasized the negative consequences of nondisclosure, this study highlights its complexity and adaptive potential in certain contexts.^23^ Past research has highlighted that nondisclosure, especially in the context of traumatic events and suicidal experiences, presents challenges that may impede emotional processing and affect adjustment outcomes.^50^^,^^51^ However, our findings demonstrate that nondisclosure patterns among adolescents are nuanced. For example, in terms of reasons for nondisclosure, self-reliance might indicate a positive coping mechanism whereby adolescents are developing independence and resilience. Similarly, the dismissal of suicidality might suggest that the adolescent no longer perceives suicidal thoughts as a present concern, indicating progress in managing their mental health. This underscores the need to examine variations in nondisclosure, a facet previously explored within other stigmatized identities but often overlooked in adolescent suicide research.

The patterns and underlying reasons for adolescent nondisclosure of suicide-related experiences to family members and peers have significant implications for developing interpersonal strategies and targeted treatments to more effectively reach these adolescents. This study found that adolescents’ nondisclosure of suicide-related experiences to their primary confidants is driven mainly by fear of negative reactions and self-reliance. Although this may be protective with peers, who may not know how to offer appropriate support, it may be less beneficial with adult family members who could connect these adolescents with care.^6^ Therefore, adult family members could focus on fostering nonjudgmental environments and promoting a culture of mutual support to reduce reasons for nondisclosure.^52^ This study also found that perpetual nondisclosers felt closer to their primary confidants despite never disclosing to them. If this finding of increased closeness is in fact linked to heightened feelings of burdensomeness, then interventions aimed at reducing perceived burdensomeness (eg, Cognitive**–**Behavioral Therapy for Suicide Prevention [CBT-SP]) could facilitate disclosure to close confidants.^53^

Future research should explore the motivations and contexts behind adolescents’ nondisclosure of suicide-related experiences to continue understanding its potential benefits and risks. Future studies should build on this research by exploring the adaptive potential of nondisclosure to better understand when and how it may benefit adolescents’ mental health outcomes. Furthermore, future research should also examine differences in disclosure to adults vs nonadult peers, as these relationships likely involve distinct dynamics and implications for mental health support. For instance, disclosures to adults may focus on seeking guidance or intervention, whereas disclosure to peers may emphasize emotional connection or relatability.^3^^,^^18^ Clinicians can use these findings to understand potential reasons for nondisclosure across contexts and to individualize their strategies to foster trust and communication both inside and outside of the therapy room, enabling adolescents to develop more effective ways to harness their family members and peers.

## CRediT authorship contribution statement

**Angela Page Spears:** Writing – review & editing, Writing – original draft, Visualization, Validation, Resources, Project administration, Methodology, Investigation, Formal analysis, Data curation, Conceptualization. **Ki Eun Shin:** Writing – review & editing, Writing – original draft, Supervision, Methodology, Formal analysis. **Christine B. Cha:** Writing – review & editing, Writing – original draft, Supervision, Resources, Project administration, Methodology, Investigation, Formal analysis, Data curation, Conceptualization.
