# Supplementary material for: Characterizing Silence: Adolescents’ Nondisclosure of Their Suicidal Thoughts and Behaviors to Their Family and Peers
Source: JAACAP Open. 2025 Feb 20;3(3):496–505. doi: 10.1016/j.jaacop.2024.12.005 (PMC12414306; doi:10.1016/j.jaacop.2024.12.005)
Supplement: Supplement 1 [file mmc1.pdf]

Reasons for Suicide-Related Nondisclosure Questionnaire

Select the reasons why you have chosen **not** to disclose your suicidal thought(s) and/or behavior(s) to your *{insert name of the person previously indicated by participant as the person they have not made a suicide disclosure to, but they have shared the most other personal information with}*.

If you are unsure about how to answer a question, please select the answer that you think is most true or you can always choose to skip questions.

	Not at all	A little	Moderately	Quite a bit	Extremely
I wasn’t ready to face my suicidal thoughts	○	○	○	○	○
I was afraid they would judge me	○	○	○	○	○
I was worried that they would think of me differently	○	○	○	○	○
I was worried that they would be angry with me	○	○	○	○	○
I didn’t want them to tell someone else	○	○	○	○	○
I was afraid that I would be hospitalized	○	○	○	○	○
I was afraid of being forced into treatment I didn’t want	○	○	○	○	○
I did not feel comfortable sharing such intimate information	○	○	○	○	○
I did not want to be viewed as weak	○	○	○	○	○
I did not want to be viewed as unstable	○	○	○	○	○
I did not want it to damage our relationship	○	○	○	○	○
I did not want to worry them	○	○	○	○	○

	Not at all	A little	Moderately	Quite a bit	Extremely
I did not want them to stop me from my future suicide plans	○	○	○	○	○
I will not experience suicidal thoughts and behaviors in my future so there was no point in disclosing	○	○	○	○	○
It is not part of who I am now	○	○	○	○	○
I have not had the chance to tell them yet	○	○	○	○	○

Powered by Qualtrics
